# Factors associated with depressive symptoms among cancer patients: a nationwide cross-sectional study

**DOI:** 10.1186/s12889-024-18898-9

**Published:** 2024-05-29

**Authors:** Xiaoqing Chen, Chaoyan Ye, Li Liu, Xiuyang Li

**Affiliations:** 1 Library, The First Affiliated Hospital, College of Medicine, Zhejiang University, Hangzhou, 310003 China; 2 Department of Big Data in Health Science, Center for Clinical Big Data and Statistics, Second Affiliated Hospital, College of Medicine, Zhejiang University, Hangzhou, 310058 China

**Keywords:** Depression, Neoplasms, Cross-sectional studies, Logistic models

## Abstract

**Objective:**

Research on factors contributing to depressive symptoms in cancer patients at a national level, encompassing a comprehensive set of variables was limited. This study aimed to address this gap by identifying the factors associated with depressive symptoms among cancer patients through a nationwide cross-sectional analysis.

**Methods:**

Various factors, including demographic, socioeconomic, behavioral patterns, general and self-rated health status, chronic conditions, dietary habits, and cancer-related factors, were examined. Data was from the National Health and Nutrition Examination Survey. Univariate and multivariate logistic regression analyses were performed to identify associated factors. The receiver-operating characteristic (ROC) curve was used to evaluate the performance of the logistic model.

**Results:**

The findings showed that five sociodemographic factors, two behavioral styles, self-rated health status, comorbid arthritis, two dietary factors and two cancer-related factors were strongly associated with depressive symptoms. Compared with those aged 20–39 years, cancer individuals aged 40–59 years (OR = 0.48, *P* < 0.05) and those 60 years or older (OR = 0.18, *P* < 0.05) had lower odds of depression. Positive factors included being never married (OR = 1.98, *P* < 0.05), widowed, divorced or separated (OR = 1.75, *P* < 0.05), unemployment (OR = 1.87, *P* < 0.05), current smoking (OR = 1.84, *P* < 0.05), inadequate sleep (OR = 1.96, *P* < 0.05), comorbid arthritis (OR = 1.79, *P* < 0.05), and poor self-rated health status (OR = 3.53, *P* < 0.05). No significant association was identified between the Healthy Eating Index 2015 and the Dietary Inflammatory Index with depression (*P* > 0.05). Shorter cancer diagnosis duration was associated with reduced odds of depression (*P* < 0.05). The logistic model had an area under the curve of 0.870 (95% CI: 0.846–0.894, *P* < 0.05).

**Conclusions:**

Cancer patients should receive enhanced family and social support while cultivating a healthy lifestyle and diet. Incorporating plenty of fruits, greens, and beans is highly recommended, along with establishing a comprehensive health management framework.

**Supplementary Information:**

The online version contains supplementary material available at 10.1186/s12889-024-18898-9.

## Introduction

Cancer is the second leading cause of death globally [1], accounting for an estimated 19.9 million new cancer cases and almost 10 million cancer-related deaths worldwide in 2022 [2]. The global incidence of cancer has been steadily increasing over the years [3], and the World Health Organization (WHO) predicts a 50% increase in cancer cases by the year 2040 [2]. This escalating cancer burden poses significant physical and economic challenges not only to individuals, families, communities, and healthcare systems but also presents substantial mental health challenges for patients. Depression was a common but often overlooked mental complication associated with cancer, impacting up to 20% of cancer patients [4, 5]. The odds of being depressed were significantly higher in cancer patients than in the general population [6]. Moreover, depressive symptoms were significantly associated with all-cause mortality, suicide, and suicidal ideation in individuals with cancer [7, 8]. Identifying factors contributing to depressive symptoms in cancer patients is crucial for developing effective interventions and support systems.

Several previous research reported the factors of depressive symptoms in cancer patients. However, the existing literature had some limitations. First, many studies focused on specific groups of cancer patients, such as hospitalization [9], employer-based health insurance claims data [10] or those undergoing chemotherapy or other treatment [11, 12]. Cancer is a disease with long-term implications. It’s important to monitor their psychological well-being throughout the entire cancer survivorship journey, not just during specific periods. Second, numerous studies were directed toward patients diagnosed with specific types of tumors, such as lung carcinoma [13, 14], head and neck cancer [15, 16], hepatocellular carcinoma [17], and breast cancer [18]. Lastly, further research is needed to investigate the impact of more individual factors on depressive symptoms in cancer patients, such as more chronic disease and dietary patterns. There was limited comprehensive research on the factors of depressive symptomatology among cancer survivors, similar to the study of Perianayagam A et al. [19]. Moreover, considering the severe burden of depression among cancer patients in recent years [20], and the limited research based on the national population, more studies involving diverse factors and samples are necessary to identify and prevent of depressive symptomatology in cancer patients.

To this end, this study investigated the association of depressive symptoms with the demographic and socioeconomic factors, behavioral patterns, general health and self-rated health status, co-existing chronic disease, dietary habits, and cancer-related factors in a cross-sectional study involving patients with cancer. This may aid in the early prevention of depression for cancer patients, thereby enhancing cancer management.

## Methods

### Study sample

This study drew upon data spanning six consecutive cycles of the National Health and Nutrition Examination Survey (NHANES) conducted between 2007 and 2018. NHANES is a nationally representative survey designed to assess the health and nutritional status of the US population. Further specifics about NHANES can be found in previously published reports [21].

Participants aged from 20 years to 80 years, without pregnancy, were included in the analysis if they had cancer and provided complete or imputable data for the depressive symptom assessment. Cancer was defined according to the participants’ self-reporting of a doctor’s diagnosis. A nine-item depression screening instrument, also called the Patient Health Questionnaire (PHQ) was administered to determine the frequency of depression symptoms over the past 2 weeks. A score of 10 or higher was indicative of potential depression [22]. In NHANES, patients who received a cancer diagnosis at the age of 80 or older were uniformly documented as “80”. And, for the 2017–2018 dataset, individuals diagnosed with cancer at an age younger than 17 were consistently recorded as “16”. Thus, these cancer survivals were excluded from the analysis due to the inability to determine the years since cancer diagnosis.

### Covariates

Information on age, sex, race, education level, marital / family income / work status, health insurance, survey cycle, drinking, smoking, sedentary behavior, serum total cholesterol (tchol), body mass index (BMI), sleep hours, comorbidities (hypertension, diabetes, asthma, arthritis, gout, congestive heart failure, coronary heart disease, angina, heart attack, stroke, emphysema, chronic bronchitis), health status, medication use, Healthy Eating Index 2015 (HEI2015), Dietary Inflammatory Index (DII), years since cancer diagnosis and cancer site was extracted from the NHANES database.

The poverty impact ratio (PIR) was used to estimate the adequacy of family income. For smoking, participants were categorized as never smokers (participants who had smoked < 100 cigarettes in their lifetime), former smokers (participants who had smoked ≥ 100 cigarettes in life but not smoking currently), or current smokers (participants who had smoked cigarettes every day or some days at the time of the survey) [23]. Sedentary behavior (< 4 h/day, 4–6 h/day, ≥ 6 h/day) was assessed based on responses to the question, “How much time do you usually spend sitting on a typical day?” [24, 25]. In the survey, serum total cholesterol was directly measured in the laboratory using enzymatic colorimetric methods. According to the standard WHO criteria, BMI was categorized into four groups (underweight BMI < 18.5 kg/m^2^, normal weight 18.5 to ≤ 24.9 kg/m^2^, overweight 25.0 ≤ 29.9 kg/m^2^, obesity ≥ 30 kg/m^2^) [26]. Sleep duration was categorized as < 7 h, 7 to 9 h, and ≥ 9 h from a question, “How much sleep do you usually get at night on weekdays or workdays?” [27]. Twelve physical comorbidities were identified based on individuals’ self-reporting diagnoses from doctors. Meantime, fasting plasma glucose greater than 125 mg/dl was also diagnosed as diabetes [28]. Health status was assessed based on self-reported general health conditions. Medication use referred to whether prescription medicines were taken in the past 30 days during the survey.

Furthermore, individual 24-hour dietary recall interviews were conducted to collect food intake data in the NHANES. The HEI2015 was calculated to evaluate the overall diet quality with the Dietary Guidelines for Americans. HEI2015 encompasses 13 components: total vegetables, greens and beans, total fruits, whole fruits, whole grains, dairy, total protein foods, seafood and plant proteins, fatty acids, refined grains, sodium, saturated fats, and added sugars. A higher score on the HEI2015 indicates a greater level of adherence to these guidelines [29]. The Dietary Inflammatory Index (DII) was employed to evaluate an individual’s inflammatory potential of the diet [30]. In this study, a total of 28 food parameters were utilized to calculate the DII score. These parameters included alcohol, energy, protein, carbohydrates, fiber, total fat, saturated fat, monounsaturated fatty acids, polyunsaturated fatty acids, cholesterol, β-carotene, thiamin, riboflavin, niacin, folic acid, Vitamin A/B6/B12/C/D/E, magnesium, iron, zinc, selenium, caffeine, and N3 and N6 fatty acids. HEI2015, HEI2015 component scores and DII scores were calculated by the dietary-index package [31]. HEI2015 scores were divided into two groups with a line of 60 [23], and DII was divided into four quartiles [32].

### Statistical analysis method

Data were analyzed with R 4.3.1 and SPSS 26.0.0. Univariate analysis and multivariate logistic regression analysis were performed to identify factors associated with depressive symptoms. In the univariate analysis, the chi-square test was applied to categorical count data, while the Wilcoxon rank-sum test was utilized for continuous variables due to their non-normal distribution. Significant variables identified in the univariate analysis were incorporated as independent variables in the logistic regression. Additionally, the receiver-operating characteristic (ROC) curve was used to evaluate the performance of the logistic model. The significance threshold was established at *P* ≤ 0.05.

## Results

### Basic characteristic

Table 1 presented the basic characteristics of the 2,290 cancer survivors, including demographic and socioeconomic factors, behavioral factors, general health, self-rated health status, comorbidities, dietary factors, and cancer-related factors. Of these subjects, 265 individuals had depressive symptoms, and 2,025 individuals had no depressive symptoms. Most tumor patients were females (53.89%), aged 60 years or older (66.46%), had a high school education or equivalent (53.69%), were non-Hispanic white people (61.88%), were either married or living with partner (62.24%), were not working or looking for work (63.78%), had health insurance (92.56%), drinking (88.52%), and had hypertension(56.19%) and arthritis (50.55%).

**Table 1:** Basic characteristics of the samples and unvariable analysis of depressive symptoms among cancer patients

Domain	Variables	ALL	No	Yes	Statistics	*P*
**Survey wave**	Wave (%)				5.73	0.334
	2007–2008	417 (18.21)	358 (17.68)	59 (22.26)		
	2009–2010	424 (18.52)	383 (18.91)	41 (15.47)		
	2011–2012	316 (13.80)	277 (13.68)	39 (14.72)		
	2013–2014	384 (16.77)	336 (16.59)	48 (18.11)		
	2015–2016	382 (16.68)	343 (16.94)	39 (14.72)		
	2017–2018	367 (16.03)	328 (16.20)	39 (14.72)		
**Demographic factors**	Age (%)				58.94	< 0.001
	20-39y	182 (7.95)	140 (6.91)	42 (15.85)		
	40-59y	586 (25.59)	486 (24.00)	100 (37.74)		
	>=60y	1522 (66.46)	1399 (69.09)	123 (46.42)		
	Sex (%)				37.46	< 0.001
	Female	1234 (53.89)	1044 (51.56)	190 (71.70)		
	Male	1056 (46.11)	981 (48.44)	75 (28.30)		
	Race (%)				15.58	0.001
	Mexican American	180 (7.86)	145 (7.16)	35 (13.21)		
	Non-Hispanic Black	381 (16.64)	340 (16.79)	41 (15.47)		
	Non-Hispanic White	1417 (61.88)	1272 (62.81)	145 (54.72)		
	Others	312 (13.62)	268 (13.23)	44 (16.60)		
	Marital status (%)				47.10	< 0.001
	Married / Living with partner	1424 (62.24)	1310 (64.76)	114 (43.02)		
	Never married	171 (7.47)	141 (6.97)	30 (11.32)		
	Widowed / Divorced/Separated	693 (30.29)	572 (28.27)	121 (45.66)		
**Socioeconomic factors**	Education (%)				61.77	< 0.001
	College or above	616 (26.91)	586 (28.94)	30 (11.36)		
	High school or equivalent	1229 (53.69)	1086 (53.63)	143 (54.17)		
	Less than high school	444 (19.40)	353 (17.43)	91 (34.47)		
	PIR (%)				133.04	< 0.001
	<1.3	555 (24.24)	420 (20.74)	135 (50.94)		
	1.3–3.5	782 (34.15)	707 (34.91)	75 (28.30)		
	>3.5	772 (33.71)	740 (36.54)	32 (12.08)		
	Unknown	181 (7.90)	158 (7.80)	23 (8.68)		
	Work type (%)				25.96	< 0.001
	Working / With a job or business	828 (36.22)	770 (38.10)	58 (21.89)		
	Not working / looking	1458 (63.78)	1251 (61.90)	207 (78.11)		
	Health insurance = yes (%)	2116 (92.56)	1891 (93.48)	225 (85.55)	20.09	< 0.001
**Behavioral health factors**	Drinking = yes (%)	2027 (88.52)	1798 (88.79)	229 (86.42)	1.08	0.299
	Smoke (%)				95.91	< 0.001
	Never smokers	1015 (44.34)	929 (45.90)	86 (32.45)		
	Former smokers	846 (36.96)	775 (38.29)	71 (26.79)		
	Current smokers	428 (18.70)	320 (15.81)	108 (40.75)		
	Sedentary behavior (%)				4.00	0.135
	<240	513 (22.49)	443 (21.94)	70 (26.72)		
	[240,360)	582 (25.52)	525 (26.00)	57 (21.76)		
	>=360	1186 (51.99)	1051 (52.06)	135 (51.53)		
	Sleep (%)				43.54	< 0.001
	[7 h,9 h)	1190 (52.24)	1102 (54.61)	88 (33.85)		
	<7 h	777 (34.11)	645 (33.96)	132 (50.77)		
	>=9 h	311 (13.65)	271 (13.43)	40 (15.38)		
**General health factors**	Tchol^a^	191 (57)	191.00 (55.50)	196.00 (62.75)	-1.74	0.081
	BMI (%)				5.61	0.132
	Normal-weight	526 (23.33)	472 (23.62)	54 (21.01)		
	Obesity	955 (42.35)	831 (41.59)	124 (48.25)		
	Overweight	745 (33.04)	671 (33.58)	74 (28.79)		
	Under-weight	29 (1.29)	24 (1.20)	5 (1.95)		
	Medication use = yes (%)	1965 (85.88)	1733 (85.66)	232 (87.55)	0.54	0.463
**Self-rated health status**	Health status (%)				334.64	< 0.001
	Fair	518 (22.62)	409 (20.20)	109 (41.13)		
	Good	1609 (70.26)	1532 (75.65)	77 (29.06)		
	Poor	163 (7.12)	84 (4.15)	79 (29.81)		
**Comorbidities**	Hypertension = yes (%)	1285 (56.19)	1121 (55.44)	164 (61.89)	3.70	0.054
	Diabetes = yes (%)	555 (24.98)	478 (24.31)	77 (30.08)	3.72	0.054
	Asthma = yes (%)	406 (17.74)	321 (15.87)	85 (32.08)	41.07	< 0.001
	Arthritis = yes (%)	1155 (50.55)	978 (48.39)	177 (67.05)	31.76	< 0.001
	Gout = yes (%)	216 (9.44)	190 (9.39)	26 (9.85)	0.02	0.899
	Congestive heart failure = yes (%)	137 (6.00)	106 (5.24)	31 (11.88)	16.86	< 0.001
	Coronary heart disease = yes (%)	172 (7.54)	138 (6.84)	34 (12.98)	11.66	0.001
	Angina = yes (%)	105 (4.60)	81 (4.01)	24 (9.13)	12.72	< 0.001
	Heart attack = yes (%)	179 (7.83)	141 (6.97)	38 (14.39)	16.80	< 0.001
	Stroke = yes (%)	170 (7.43)	124 (6.13)	46 (17.42)	41.71	< 0.001
	Emphysema = yes (%)	125 (5.47)	96 (4.75)	29 (10.98)	16.41	< 0.001
	Chronic bronchitis = yes (%)	243 (10.65)	191 (9.47)	52 (19.62)	24.29	< 0.001
**Dietary factors**	HEI2015 (%)				10.15	0.001
	<60	1628 (71.09)	1417 (69.98)	211 (79.62)		
	>=60	662 (28.91)	608(30.02)	54 (20.37)		
	HEI2015_total fruit^b^	2.04 (5.00)	2.21 (4.95)	0.37 (4.12)	-4.89	< 0.001
	HEI2015_ whole fruits^b^	1.81 (5.00)	2.15 (5.00)	0.00 (5.00)	-4.02	< 0.001
	HEI2015_total vegetables^b^	3.33 (4.96)	3.41 (3.16)	2.84 (3.73)	-3.70	< 0.001
	HEI2015_greens and beans^b^	0.00 (4.06)	0.00 (4.46)	0.00 (1.17)	-2.88	0.004
	HEI2015_total protein foods^c^	5.00 (1.09)	5.00 (0.96)	5.00 (1.58)	-3.77	< 0.001
	HEI2015_seafood and plant proteins^c^	1.79 (5.00)	2.03 (5.00)	0.52 (5.00)	-2.99	0.003
	HEI2015_whole grains^c^	1.30 (5.00)	1.45 (5.11)	0.00 (3.44)	-3.81	< 0.001
	HEI2015_dairy^b^	4.62 (6.19)	4.65 (6.23)	4.28 (5.81)	-1.75	0.08
	HEI2015_fatty acids^d^	4.76 (7.36)	4.76 (7.24)	4.81 (8.36)	-0.78	0.436
	HEI2015_refined grains^c^	7.47 (6.25)	7.45 (6.18)	7.69 (6.78)	-0.17	0.867
	HEI2015_sodium^d^	4.06 (6.8)	3.95 (6.62)	5.20 (7.34)	-3.93	< 0.001
	HEI2015_added sugars^e^	7.94 (5.51)	8.04 (5.19)	6.85 (7.82)	-4.58	< 0.001
	HEI2015_saturated fats^e^	6.17 (6.34)	6.15 (6.35)	6.62 (6.26)	-1.09	0.274
	DII (%)				50.10	< 0.001
	Q1: -3.99 ~ 0.364	573 (25.02)	526 (25.98)	47 (17.74)		
	Q2: 0.364 ~ 1.810	572 (24.98)	527 (26.02)	45 (16.98)		
	Q3: 1.810 ~ 3.079	572 (24.98)	511 (25.23)	61 (23.02)		
	Q4: 3.079 ~ 5.270	573 (25.02)	461 (22.77)	112 (42.26)		
**Cancer-related factors**	Years since cancer diagnosis (%)				13.27	0.001
	>=10	992 (43.49)	857 (42.43)	135 (51.72)		
	5 ~ 9	561 (24.59)	519 (25.69)	42 (16.09)		
	0 ~ 4	728 (31.92)	644 (31.88)	84 (32.18)		
	Cancer site (%)				98.28	< 0.001
	Stomach	14 (0.62)	9 (0.45)	5 (1.93)		
	Lung	47 (2.07)	42 (2.08)	5 (1.93)		
	Colorectal	140 (6.16)	123 (6.10)	17 (6.56)		
	Hepatobiliary and pancreatic	19 (0.84)	11 (0.55)	8 (3.09)		
	Melanoma	136 (5.98)	126 (6.25)	10 (3.86)		
	Skin non-melanoma	322 (14.16)	304 (15.09)	18 (6.95)		
	Skin unknown type	161 (7.08)	144 (7.15)	17 (6.56)		
	Breast	349 (15.35)	307 (15.24)	42 (16.22)		
	Prostate	336 (14.78)	316 (15.68)	20 (7.72)		
	Bladder	49 (2.15)	43 (2.13)	6 (2.32)		
	Kidney	41 (1.80)	37 (1.84)	4 (1.54)		
	Cervix	174 (7.65)	136 (6.75)	38 (14.67)		
	Uterine	102 (4.49)	77 (3.82)	25 (9.65)		
	Ovary	59 (2.59)	45 (2.23)	14 (5.41)		
	Hematological	90 (3.96)	82 (4.17)	8 (3.09)		
	Thyroid	52 (2.29)	44 (2.18)	8 (3.09)		
	Other	183 (8.05)	169 (8.39)	14 (5.41)		

Count variables were represented using case numbers and percentages, continuous variables were described using the median (interquartile range)

Units of continuous variables: a, mg/dL; b, cup equivalents; c, ounce equivalents; d, g; e, % of energy

PIR = poverty impact ratio, Tchol = total cholesterol, BMI = body mass index, HEI2015 = Healthy Eating Index 2015, DII = Dietary Inflammatory Index

In addition, 51.99% of the samples had a sedentary behavior with a sitting time of 6 h or more, 52.24% had sufficient sleep time, and 70.26% self-assessed their health status as good. The HEI2015 ranged from 14.40 to 93.77 and DII scores ranged from − 3.99 to 5.27. 85.88% of the cancer survivors were using prescription medications. The three most common types of cancer were breast cancer (15.35%), prostate cancer (14.78), and non-melanoma skin cancer (14.16%). 43.49% of the patients had been diagnosed with cancer for over 10 years.

### Univariate analysis

The results of univariate analysis (Table 1) indicated that all demographic and socioeconomic factors in this study were statistically associated with depressive symptoms in cancer patients (*P* < 0.05).

For behavioural health factors, there were significant differences between those with and without depressive symptoms in smoking status (χ^2^ = 95.91, *P* < 0.001) and sleep duration (χ^2^ = 43.54, *P* < 0.001). Moreover, self-rated health status and nine physical comorbidities were statistically correlated with depressive symptoms in cancer patients (*P* < 0.05).

In terms of dietary factors, HEI2015 score (χ^2^ = 10.15, *P* = 0.001) and DII (χ^2^ = 50.10, *P* < 0.001) of cancer patients with depressive symptoms were markedly different from those without depressive symptoms. For detailed dietary components, nine components excluding dairy (z =−1.75, *P* > 0.05), fatty acids (z =−0.78, *P* > 0.05), refined grains (z =−0.17, *P* > 0.05) and saturated fats (z =−1.09, *P* > 0.05) had statistically significant effects on depression.

Finally, two cancer-related factors, years since cancer diagnosis (χ^2^ = 13.27, *P* = 0.001) and cancer site (χ^2^ = 98.28, *P* < 0.001) were also found to be statistically different.

### Multivariable analysis

Thirty-three variables that exhibited significant differences in univariate analysis were screened and included in the multivariate regression analysis to determine potential associated factors for depressive symptoms. There was no multicollinearity among the chosen variables with the evidence of all variance inflation factor (VIF) values < 5 (Supplementary material 1). The multivariable analysis results were shown in Table 2.

**Table 2:** Multivariable analysis of depressive symptoms among cancer patients

Domain	Variables		b	SE	Waldχ^2^	*P*-value	OR	95% CI
**Demographic factors**	Age	20-39y	Ref					
		40-59y	-0.733	0.297	6.109	**0.013**	0.48	**(0.27,0.86)**
		>=60y	-1.707	0.338	25.472	**< 0.001**	0.18	**(0.09,0.35)**
	Sex	Female	Ref					
		Male	-0.652	0.259	6.362	**0.012**	0.52	**(0.31,0.87)**
	Race	Mexican american	Ref					
		Non-hispanic black	-0.957	0.345	7.685	**0.006**	0.38	**(0.20,0.76)**
		Non-hispanic white	-0.632	0.3	4.439	**0.035**	0.53	**(0.30,0.96)**
		Others	-0.385	0.325	1.403	0.236	0.68	(0.36,1.29)
	Marital status	Married/living with partner	Ref					
		Never married	0.685	0.292	5.521	**0.019**	1.98	**(1.12,3.51)**
		Widowed/divorced/separated	0.561	0.185	9.197	**0.002**	1.75	**(1.22,2.52)**
**Socioeconomic factors**	Education	College or above	Ref					
		High school or equivalent	-0.04	0.261	0.024	0.877	0.96	(0.58,1.60)
		Less than high school	-0.119	0.309	0.149	0.699	0.89	(0.48,1.63)
	PIR	< 1.3	Ref					
		1.3–3.5	-0.147	0.204	0.52	0.471	0.86	(0.58,1.29)
		> 3.5	-0.522	0.287	3.325	0.068	0.59	(0.34,1.04)
		Unknown	-0.048	0.33	0.021	0.883	0.95	(0.50,1.82)
	Work type	Working/with a job or business	Ref					
		Not working/looking	0.623	0.213	8.546	**0.003**	1.87	**(1.23,2.83)**
	Health insurance	No	Ref					
		Yes	-0.348	0.265	1.73	0.188	0.71	(0.42,1.19)
**Behavioral health factors**	Smoke	Never smokers	Ref					
		Former smokers	0.208	0.211	0.973	0.324	1.23	(0.81,1.86)
		Current smokers	0.611	0.238	6.6	**0.01**	1.84	**(1.16,2.94)**
	Sleep	[7 h,9 h)	Ref					
		< 7 h	0.673	0.183	13.519	**< 0.001**	1.96	**(1.37,2.80)**
		>=9 h	0.299	0.257	1.358	0.244	1.35	(0.82,2.23)
**Self-rated health status**	Health status	Fair	Ref					
		Good	-1.325	0.192	47.661	**< 0.001**	0.27	**(0.18,0.39)**
		Poor	1.262	0.251	25.31	**< 0.001**	3.53	**(2.16,5.78)**
**Comorbidities**	Asthma	No	Ref					
		Yes	0.242	0.209	1.342	0.247	1.27	(0.85,1.92)
	Arthritis	No	Ref					
		Yes	0.584	0.185	9.944	**0.002**	1.79	**(1.25,2.58)**
	Congestive heart failure	No	Ref					
		Yes	0.426	0.332	1.648	0.199	1.53	(0.80,2.93)
	Coronary heart disease	No	Ref					
		Yes	0.203	0.336	0.365	0.546	1.23	(0.64,2.36)
	Angina	No	Ref					
		Yes	-0.417	0.376	1.23	0.267	0.66	(0.32,1.38)
	Heart attack	No	Ref					
		Yes	0.01	0.327	0.001	0.975	1.01	(0.53,1.92)
	Stroke	No	Ref					
		Yes	0.352	0.266	1.746	0.186	1.42	(0.84,2.4)
	Emphysema	No	Ref					
		Yes	-0.021	0.35	0.004	0.952	0.98	(0.49,1.95)
	Chronic bronchitis	No	Ref					
		Yes	-0.274	0.258	1.133	0.287	0.76	(0.46,1.26)
**Dietary factors**	HEI2015	< 60	Ref					
		>=60	0.052	0.28	0.034	0.853	1.05	(0.61,1.82)
	HEI2015_total fruit		-0.17	0.077	4.788	**0.029**	0.84	**(0.73,0.98)**
	HEI2015_ whole fruit		0.095	0.072	1.742	0.187	1.1	(0.96,1.27)
	HEI2015_total vegetables		0.063	0.055	1.311	0.252	1.07	(0.96,1.19)
	HEI2015_greens and beans		-0.129	0.05	6.646	**0.01**	0.88	**(0.80,0.97)**
	HEI2015_total protein foods		-0.108	0.067	2.575	0.109	0.9	(0.79,1.02)
	HEI2015_seafood and plant proteins		0.053	0.045	1.408	0.235	1.06	(0.97,1.15)
	HEI2015_whole grain		-0.007	0.028	0.064	0.8	0.99	(0.94,1.05)
	HEI2015_sodium		0.051	0.028	3.25	0.071	1.05	(1.00,1.11)
	HEI2015_added sugar		0.006	0.027	0.047	0.829	1.01	(0.95,1.06)
	DII	Q1	Ref					
		Q2	-0.375	0.266	1.99	0.158	0.69	(0.41,1.16)
		Q3	-0.458	0.277	2.746	0.098	0.63	(0.37,1.09)
		Q4	-0.084	0.276	0.092	0.761	0.92	(0.54,1.58)
**Cancer-related factors**	Years since cancer diagnosis	>=10	Ref					
		5 ~ 9	-0.669	0.231	8.405	**0.004**	0.51	**(0.33,0.81)**
		0 ~ 4	-0.402	0.203	3.929	**0.047**	0.67	**(0.45,0.995)**
	Cancer site	Stomach	Ref					
		Lung	-1.668	0.907	3.381	0.066	0.19	(0.03,1.12)
		Colorectal	-1.232	0.793	2.409	0.121	0.29	(0.06,1.38)
		Hepatobiliary and pancreatic	0.186	1.005	0.034	0.853	1.21	(0.17,8.64)
		Melanoma	-1.459	0.83	3.086	0.079	0.23	(0.05,1.18)
		Skin non-melanoma	-1.719	0.795	4.678	**0.031**	0.18	**(0.04,0.85)**
		Skin unknown type	-1.071	0.802	1.782	0.182	0.34	(0.07,1.65)
		Breast	-1.417	0.768	3.406	0.065	0.24	(0.05,1.09)
		Prostate	-1.074	0.792	1.838	0.175	0.34	(0.07,1.61)
		Bladder	-1.428	0.922	2.397	0.122	0.24	(0.04,1.46)
		Kidney	-1.675	0.978	2.933	0.087	0.19	(0.03,1.27)
		Cervix	-1.888	0.792	5.688	**0.017**	0.15	**(0.03,0.71)**
		Uterine	-1.067	0.798	1.79	0.181	0.34	(0.07,1.64)
		Ovary	-1.419	0.847	2.808	0.094	0.24	(0.05,1.27)
		Hematological	-1.946	0.868	5.02	**0.025**	0.14	**(0.03,0.78)**
		Thyroid	-1.159	0.853	1.845	0.174	0.31	(0.06,1.67)
		Other	-1.753	0.806	4.73	**0.03**	0.17	**(0.04,0.84)**

PIR = poverty impact ratio, HEI2015 = Healthy Eating Index 2015, DII = Dietary Inflammatory Index, OR = odds ratio, CI = confidence interval

### Demographic and socioeconomic factors

For cancer patients, individuals aged 40–59 years (OR = 0.48, 95%CI: 0.27–0.86) and those ≥ 60 years (OR = 0.18, 95%CI: 0.09–0.35) had lower prevalence odds of depression compared to those aged 20–39 years. Males had lower odds compared to females (OR = 0.52, 95%CI: 0.31–0.87). The result showed a statistical association between ethnicity and depression. Additionally, the prevalence odds of experiencing depressive symptoms for cancer survivals who have never been married (OR = 1.98, 95%CI: 1.12–3.51), widowed, divorced, or separated (OR = 1.75, 95%CI: 1.22–2.52) were higher compared to those who had been married or living with a partner. Cancer individuals without employment (OR = 1.87, 95%CI: 1.23–2.83) had higher prevalence odds of depressive symptoms than those with employment.

### Behavioral health factors

Table 2 showed that two bad lifestyle behaviors, current smoking (OR = 1.84, 95%CI: 1.16–2.94) and sleeping less than 7 h (OR = 1.96, 95%CI: 1.37–2.80) were positively associated with depressive symptoms.

### Self-rated health status and comorbidities

Furthermore, cancer individuals who rated their health as good had lower prevalence odds of depressive symptoms (OR = 0.27, 95%CI: 0.18–0.39), while the opposite was true for those with poor self-rated health status (OR = 3.53, 95%CI: 2.16–5.78). Cancer patients coexisting with arthritis increased the prevalence odds of depressive symptoms (OR = 1.79, 95%CI: 1.25–2.58).

### Dietary factors

Interestingly, no significant association was identified between HEI2015 and DII with depression (*P* > 0.05). However, an increased intake of total fruits (OR = 0.84, 95%CI: 0.73–0.98), as well as greens and beans (OR = 0.88, 95%CI: 0.80–0.97), may help reduce the odds of depression in cancer patients.

### Cancer-related factors

In addition, the prevalence odds of depression were lower in individuals diagnosed with cancer within 0–4 years (OR = 0.67, 95%CI: 0.45–0.995) and 5–9 years (OR = 0.51, 95%CI: 0.33–0.81) compared to those who have been living with cancer for over 10 years. Besides, Compared to individuals with stomach tumors, those with non-melanoma skin (OR = 0.18, 95%CI: 0.04–0.85), cervix tumors (OR = 0.15, 95%CI: 0.03–0.71), or with hematological cancers (OR = 0.14, 95%CI: 0.03–0.78) have lower odds of experiencing depressive symptoms.

### ROC curve

**Fig. 1:**
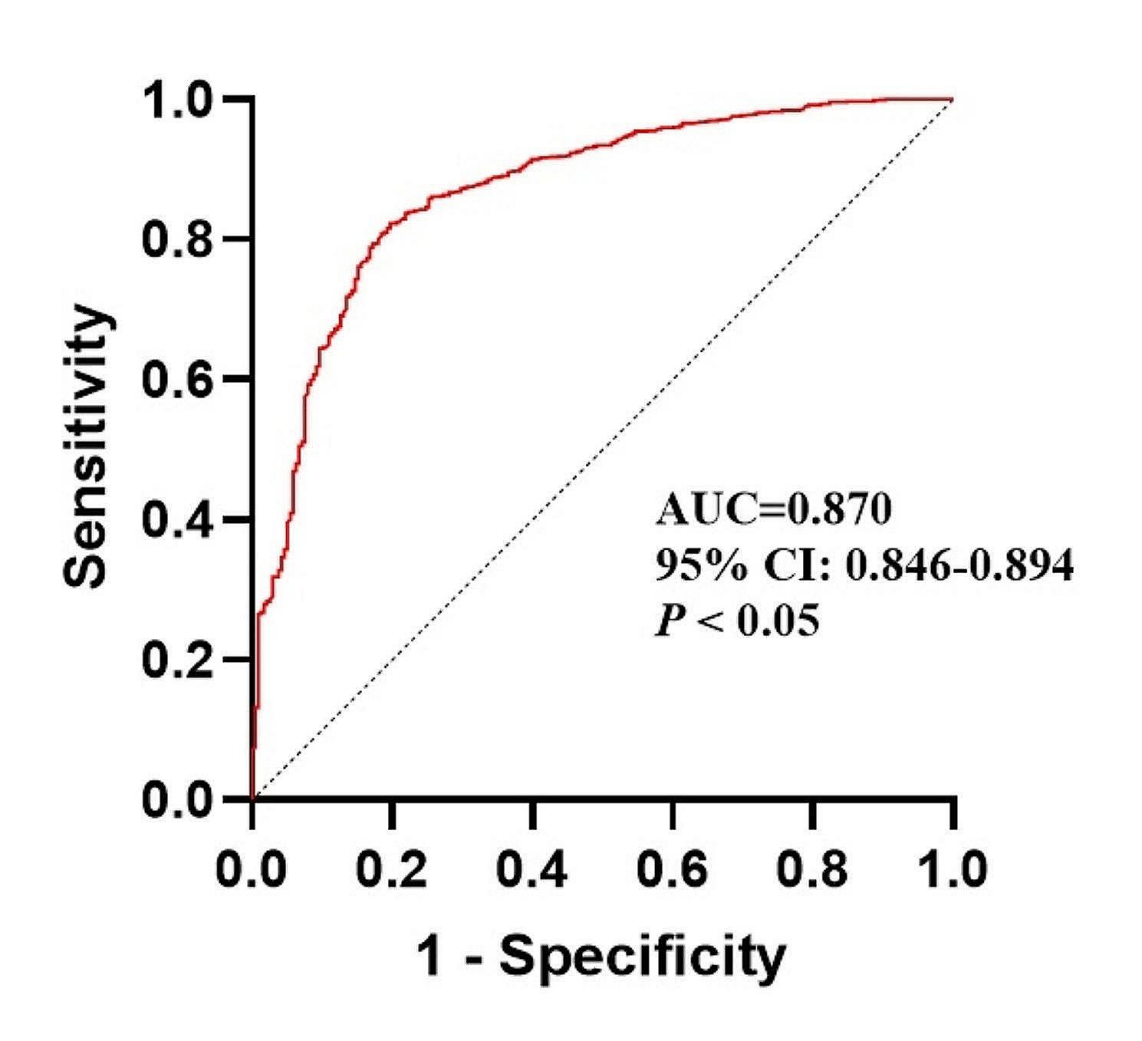
ROC curve and AUC of the multivariate logistic models

The combined area under (AUC) the curve of the multivariable, as shown in Fig. 1, demonstrated that the model had high accuracy (AUC = 0.870, 95% CI: 0.846–0.894). Therefore, the regression equation model for depressive symptoms among cancer patients was obtained by the above regression analysis (Supplementary material 2).

## Discussion

This study presented a thorough investigation into the factors influencing depressive symptoms among cancer patients. The research encompassed a wide array of aspects, including demographics, socioeconomic status, lifestyle habits, comorbidities, health status, and cancer-related factors. The comprehensive study has the potential to offer insights for cancer management and help prevent and alleviate depressive symptoms in cancer patients, thereby enhancing their holistic quality of life.

The result showed that age, sex, race and marital status were strongly associated with depressive symptoms for patients with cancer, similar to the findings of previous studies [33–35]. Higher odds of depression symptoms among young unmarried female cancer patients may be because of lacking social support and family care [36, 37], changes in physical appearance, and concerns about the future. Notably, prior research investigating the connection between income status and depression in cancer patients has produced inconsistent findings [12]. This study demonstrated that there was no correlation between family income and depression among cancer patients. Possible reasons for these differences may stem from variations in the criteria used to categorize income across studies, as well as potential biases introduced by participants’ self-reported data.

Depression is affected by many comorbidities, such as hypertension, diabetes, cardiovascular diseases, bone diseases, injury, organ related diseases [19, 38]. Among the comorbidities included in this study, only arthritis was found to be positively associated with depressive symptoms in cancer patients. There were currently few studies on the relationship between arthritis and depression symptoms in cancer patients. One study focusing on rheumatoid arthritis (RA) patients also showed that individuals with both cancers and RA had higher odds of experiencing depressive symptoms [39]. And, Petrova D et al. found that, for short-term cancer patients who have been diagnosed with tumors within 5 years, arthritis may increase the odds of depression [40]. Arthritis can cause significant pain disability, and physical limitations, which can lead to feelings of frustration, anxiety, and depression. Mental and arthritis multimorbidity could increase the odds of mortality [41]. Managing arthritis in cancer patients requires a comprehensive approach that takes into account both the physical and mental aspects of the conditions. Alongside pharmacological treatments, it is important to pay attention to the emotional aspects of arthritis in cancer patients.

A prospective cohort study in India revealed that poor self-rated health was associated with higher odds of depression [19]. This study also found that self-rated health status significantly influenced the prevalence odds of depressive symptoms in cancer patients. It was observed that tumor patients who perceived themselves to be in good health had lower prevalence odds of depression. Conversely, those who rated their health status as poor had higher prevalence odds of depressive symptoms. This suggests that subjective perceptions of health play a crucial role in shaping the psychological outcomes of cancer patients, particularly depression. Furthermore, individuals who rated their health poorly experienced a diminished overall quality of life [42]. Additionally, research revealed a higher mortality rate among cancer survivors with poor self-rated health [43]. These highlight the potential implications of subjective health perceptions on long-term outcomes for individuals facing cancer.

Moreover, this study identified that short sleep duration could increase the prevalence odds of depressive symptoms, further corroborating prior studies [33]. Similar finding was confirmed in one study specifically conducted for breast cancer patients [44]. However, a study focused on gastrointestinal cancer patients produced contrasting results, and found no correlation between sleep duration and depression [45]. The discrepancies may be attributed to variations in the criteria used to measure sleep duration and diagnose depression, as well as differences in the study samples. Meta-analysis revealed a strong association between dietary patterns and the prevalence of depression [46, 47]. However, it is crucial to highlight that the findings differed when examining a subgroup of cancer patients. No correlation was found between HEI, DII and depressive outcomes in cancer patients. One prior prospective study [48] indicated that there was no significant association between DII and depressive symptoms in the overall sample population. However, through more in-depth subgroup analysis of different populations, the research reveals a correlation. DII may not be a suitable tool for all general population [49]. This study found no significant association between DII and depressive symptoms in the entire cancer sample population, thus necessitating further subgroup analysis for a deeper understanding of this relationship. Furthermore, higher consumption of total fruit, greens and beans can reduce the prevalence of depressive symptoms in cancer patients. Lifestyle plays a crucial role in health promotion. Embracing healthy dietary habits, participating in regular physical activity, and prioritizing high-quality sleep are pivotal for preventing suboptimal health status [50]. Cancer patients should cultivate positive lifestyle habits and improve their overall well-being.

These findings lay the groundwork for the development of more targeted and effective psychological intervention strategies, ultimately enhancing the overall quality of life for cancer patients. Advocating for a holistic approach to psychological health management in cancer care, and establishing a comprehensive health management model that integrates various factors, create a more supportive and resilient healthcare ecosystem for individuals grappling with cancer.

There were still some limitations in our study: (1) Using a screening questionnaire instead of diagnostic criteria to identify depression. (2) This study was a cross-sectional analysis without prospective outcomes. (3) This study lacked laboratory and imaging data about cancer, and thus could not fully reflect the clinical characteristics of cancer patients. (4) The comorbidities were self-reported. (5) Considering the substantial amount of missing data, this study did not incorporate variables such as television viewing time, computer usage, and levels of physical activity. (6) For dietary patterns, there are various indices to assess dietary quality, such as the alternate healthy eating index, dietary approaches to stop hypertension, and cancer prevention score recommended by the American Cancer Society. However, this study did not incorporate all of these metrics.

## Conclusions

This study highlighted the increased susceptibility to depressive symptoms among younger female cancer patients, emphasizing key factors like marital status, employment status, current smoking, inadequate sleep, comorbid arthritis, and poor self-rated health status. It stressed the need for targeted psychological support. Notably, patients diagnosed with cancer for a shorter duration had lower odds of depressive symptoms. Variations in depressive prevalence by cancer sites highlighted the necessity of tailored interventions. Unexpectedly, no correlations were found between DII and HEI2015 with depression in cancer patients in this nationwide survey. Moreover, the study suggested a comprehensive psychosexual intervention model integrating various demographic, socioeconomic, behavioral, and health-related factors should be developed for cancer survival. Emphasizing the importance of strengthening social support networks, promoting healthy lifestyle habits, and encouraging dietary choices rich in fruits, greens, and beans forms the cornerstone of this approach.

### Electronic supplementary material

Below is the link to the electronic supplementary material.

Supplementary Material 1

Supplementary Material 2

## Data Availability

More information about the NHANES can be obtained at: http://www.cdc.gov/nhanes.

## References

[1] WHO, Cancer. https://www.who.int/health-topics/cancer (2022). Accessed 29 Nov 2023.

[2] Ferlay J, Ervik M, Lam F, Laversanne M, Colombet M, Mery L, Piñeros M, Znaor A, Soerjomataram I, Bray F. Global Cancer Observatory: Cancer Today. Lyon, France: International Agency for Research on Cancer. https://gco.iarc.fr/today (2024). Accessed 22 Apri 2024.

[3] Zhao J, Xu L, Sun J, Song M, Wang L, Yuan S, Zhu Y, Wan Z, Larsson S, Tsilidis K, Dunlop M, Campbell H, Rudan I, Song P, Theodoratou E, Ding K, Li X (2023). Global trends in incidence, death, burden and risk factors of early-onset cancer from 1990 to 2019. BMJ Oncol.

[4] Pitman A, Suleman S, Hyde N, Hodgkiss A (2018). Depression and anxiety in patients with cancer. BMJ.

[5] Mitchell AJ, Chan M, Bhatti H, Halton M, Grassi L, Johansen C, Meader N (2011). Prevalence of depression, anxiety, and adjustment disorder in oncological, haematological, and palliative-care settings: a meta-analysis of 94 interview-based studies. Lancet Oncol.

[6] Hartung TJ, Brahler E, Faller H, Harter M, Hinz A, Johansen C, Keller M, Koch U, Schulz H, Weis J, Mehnert A (2017). The risk of being depressed is significantly higher in cancer patients than in the general population: prevalence and severity of depressive symptoms across major cancer types. Eur J Cancer.

[7] Wang YH, Li JQ, Shi JF, Que JY, Liu JJ, Lappin JM, Leung J, Ravindran AV, Chen WQ, Qiao YL, Shi J, Lu L, Bao YP (2020). Depression and anxiety in relation to cancer incidence and mortality: a systematic review and meta-analysis of cohort studies. Mol Psychiatry.

[8] Zaorsky NG, Zhang Y, Tuanquin L, Bluethmann SM, Park HS, Chinchilli VM (2019). Suicide among cancer patients. Nat Commun.

[9] Lu W, Pikhart H, Peasey A, Kubinova R, Pitman A, Bobak M (2020). Risk of depressive symptoms before and after the first hospitalisation for cancer: evidence from a 16-year cohort study in the Czech Republic. J Affect Disord.

[10] Akechi T, Mishiro I, Fujimoto S, Murase K (2020). Risk of major depressive disorder in Japanese cancer patients: a matched cohort study using employer-based health insurance claims data. Psychooncology.

[11] Shim EJ, Hahm BJ, Yu ES, Kim HK, Cho SJ, Chang SM, Yang JC, Kim JH (2018). Prevalence, correlates, and impact of depressive and anxiety disorder in cancer: findings from a multicenter study. Palliat Support Care.

[12] Wen S, Xiao H, Yang Y (2019). The risk factors for depression in cancer patients undergoing chemotherapy: a systematic review. Support Care Cancer.

[13] Shahedah KK, How SH, Jamalludin AR, Mohd FM, Kuan YC, Ong CK (2019). Depressive symptoms in newly diagnosed lung carcinoma: prevalence and Associated Risk factors. Tuberc Respir Dis (Seoul).

[14] Lee Y, Lin PY, Lin MC, Wang CC, Lu HI, Chen YC, Chong MY, Hung CF (2019). Morbidity and associated factors of depressive disorder in patients with lung cancer. Cancer Manag Res.

[15] Fan CY, Chao HL, Lin CS, Huang WY, Chen CM, Lin KT, Lin CL, Kao CH (2018). Risk of depressive disorder among patients with head and neck cancer: a nationwide population-based study. Head Neck.

[16] Lee Y, Lin PY, Chien CY, Fang FM (2015). Prevalence and risk factors of depressive disorder in caregivers of patients with head and neck cancer. Psychooncology.

[17] Chang CH, Chen SJ, Liu CY (2015). Risk of developing depressive disorders following Hepatocellular Carcinoma: a Nationwide Population-based study. PLoS ONE.

[18] Aguado LC, Baldwin JA, McDermott RJ, McMillan S, Martinez TD, Yampolskaya S, Vandeweerd C (2013). Risk factors associated with increased depressive symptoms among latinas diagnosed with breast cancer within 5 years of survivorship. Psychooncology.

[19] Perianayagam A, Prina M, Selvamani Y, Gudekar D, Salvi S, Varghese M, Dandona R (2022). Sub-national patterns and correlates of depression among adults aged 45 years and older: findings from wave 1 of the longitudinal ageing study in India. Lancet Psychiatry.

[20] Yan G, Zhang Q, Yan Y, Zhang Y, Li Y, Liu M, Tian W (2023). Trends in the prevalence and treatment of comorbid depression among US adults with and without cancer, 2005–2020. J Affect Disord.

[21] CDC CFDC. About the National Health and Nutrition Examination survey. https://www.cdc.gov/nchs/nhanes/about_nhanes.htm (2023). Accessed 26 Nov 2023.

[22] Kroenke K, Spitzer RL, Williams JB (2001). The PHQ-9: validity of a brief depression severity measure. J Gen Intern Med.

[23] Li Y, Xia PF, Geng TT, Tu ZZ, Zhang YB, Yu HC, Zhang JJ, Guo K, Yang K, Liu G, Shan Z, Pan A (2023). Trends in Self-reported adherence to healthy lifestyle behaviors among US adults, 1999 to March 2020. JAMA Netw Open.

[24] Han H, Cao Y, Feng C, Zheng Y, Dhana K, Zhu S, Shang C, Yuan C, Zong G (2022). Association of a healthy lifestyle with all-cause and cause-specific mortality among individuals with type 2 diabetes: a prospective study in UK Biobank. Diabetes Care.

[25] Liang Y, Liu F, Yin H, Shi X, Chen Y, Wang H, Wang Y, Bai B, Liu Y, Liu Q, Wu C, Yu X, Ma H, Geng Q (2023). Trends in unhealthy lifestyle factors in US NHANES respondents with cardiovascular disease for the period between 1999 and 2018. Front Cardiovasc Med.

[26] WHO. Body mass index (BMI). https://www.who.int/data/gho/data/indicators (2023). Accessed 29 Nov 2023.

[27] Dong L, Xie Y, Zou X (2022). Association between sleep duration and depression in US adults: a cross-sectional study. J Affect Disord.

[28] Standards of medical care in diabetes, –, 2010 (2010). Diabetes Care.

[29] Krebs-Smith SM, Pannucci TE, Subar AF, Kirkpatrick SI, Lerman JL, Tooze JA, Wilson MM, Reedy J (2018). Update of the healthy eating index: HEI-2015. J Acad Nutr Diet.

[30] Shivappa N, Steck SE, Hurley TG, Hussey JR, Hebert JR (2014). Designing and developing a literature-derived, population-based dietary inflammatory index. Public Health Nutr.

[31] Zhan JJ, Hodge RA, Dunlop AL, Lee MM, Bui L, Liang D, Ferranti EP. Dietaryindex: A User-Friendly and Versatile R Package for Standardizing Dietary Pattern Analysis in Epidemiological and Clinical Studies. bioRxiv. 2023. 10.1101/2023.08.07.548466. 10.1016/j.ajcnut.2024.08.021 PMC11600030 39182618

[32] Shivappa N, Hebert JR, Veronese N, Caruso MG, Notarnicola M, Maggi S, Stubbs B, Firth J, Fornaro M, Solmi M (2018). The relationship between the dietary inflammatory index (DII((R))) and incident depressive symptoms: a longitudinal cohort study. J Affect Disord.

[33] Zhang XM, Zhang ZB, Chen W, Wu X (2022). The association between handgrip strength and depression in cancer survivors: a cross-sectional study. BMC Geriatr.

[34] Petrova D, Ubago-Guisado E, Garcia-Retamero R, Redondo-Sanchez D, Perez-Gomez B, Catena A, Caparros-Gonzalez RA, Sanchez MJ. Allostatic load and depression symptoms in Cancer survivors: a National Health and Nutrition Examination Survey Study. Cancer Nurs 2023 [cited 1 Dec 2023]. 10.1097/NCC.0000000000001216. 10.1097/NCC.0000000000001216 36920171

[35] Endo M, Matsui K, Akaho R, Mitsui K, Yan Y, Imai Y, Ueda Y, Muto G, Deshpande GA, Terao Y, Takeda S, Saito M, Hayashi K, Nishimura K, Tanigawa T (2022). Depressive and anxiety symptoms among Japanese cancer survivors: Japan cancer survivorship research project. BMC Cancer.

[36] Springer F, Sautier L, Schilling G, Koch-Gromus U, Bokemeyer C, Friedrich M, Mehnert-Theuerkauf A, Esser P (2023). Effect of depression, anxiety, and distress screeners on the need, intention, and utilization of psychosocial support services among cancer patients. Support Care Cancer.

[37] Li Q, Liu L, Gu Z, Li M, Liu C, Wu H (2023). Sense of coherence mediates perceived social support and depressive and anxiety symptoms in cervical cancer patients: a cross-sectional study. BMC Psychiatry.

[38] Han JW, Yang HW, Bae JB, Oh DJ, Moon DG, Lim E, Shin J, Kim BJ, Lee DW, Kim JL, Jhoo JH, Park JH, Lee JJ, Kwak KP, Lee SB, Moon SW, Ryu SH, Kim SG, Kim KW (2023). Shared Risk factors for depressive disorder among older adult couples in Korea. JAMA Netw Open.

[39] Lin MC, Guo HR, Lu MC, Livneh H, Lai NS, Tsai TY (2015). Increased risk of depression in patients with rheumatoid arthritis: a seven-year population-based cohort study. Clin (Sao Paulo).

[40] Petrova D, Catena A, Rodriguez-Barranco M, Redondo-Sanchez D, Bayo-Lozano E, Garcia-Retamero R, Jimenez-Moleon JJ, Sanchez MJ. Physical comorbidities and depression in recent and long-term Adult Cancer survivors: NHANES 2007–2018. Cancers (Basel) 2021; 13. 10.3390/cancers13133368 PMC8268421 34282756

[41] Fan J, Sun Z, Yu C, Guo Y, Pei P, Yang L, Chen Y, Du H, Sun D, Pang Y, Zhang J, Gilbert S, Avery D, Chen J, Chen Z, Lyu J, Li L (2022). Multimorbidity patterns and association with mortality in 0.5 million Chinese adults. Chin Med J (Engl).

[42] Chien CH, Chuang CK, Liu KL, Wu CT, Pang ST, Chang YH (2021). Health-Related Quality of Life and its Associated factors in prostate Cancer patients who receive androgen deprivation therapy. Cancer Nurs.

[43] Chavan PP, Kedia SK, Mzayek F, Ahn S, Yu X (2021). Impact of self-assessed health status and physical and functional limitations on healthcare utilization and mortality among older cancer survivors in US. Aging Clin Exp Res.

[44] Liu L, Bao H, Wang F, Yu L, Cong S, Zhou F, Xiang Y, Huang S, Zheng C, Fang L, Wang L, Yu Z (2023). Depressive symptoms and sleep duration as risk factors for breast Cancer - China, 2020. China CDC Wkly.

[45] de Sousa DE, de Carli MN, Fernandes RC, Trindade DB, Laviano A, Pichard C, Pimentel GD (2020). Are depression and anxiety disorders associated with adductor pollicis muscle thickness, sleep duration, and protein intake in cancer patients?. Exp Gerontol.

[46] Lassale C, Batty GD, Baghdadli A, Jacka F, Sanchez-Villegas A, Kivimaki M, Akbaraly T (2019). Healthy dietary indices and risk of depressive outcomes: a systematic review and meta-analysis of observational studies. Mol Psychiatry.

[47] Li Y, Lv MR, Wei YJ, Sun L, Zhang JX, Zhang HG, Li B (2017). Dietary patterns and depression risk: a meta-analysis. Psychiatry Res.

[48] Adjibade M, Andreeva VA, Lemogne C, Touvier M, Shivappa N, Hebert JR, Wirth MD, Hercberg S, Galan P, Julia C, Assmann KE, Kesse-Guyot E (2017). The inflammatory potential of the Diet is Associated with depressive symptoms in different subgroups of the General Population. J Nutr.

[49] Azarmanesh D, Pearlman J, Carbone ET, DiNatale JC, Bertone-Johnson ER. Construct validation of the Dietary Inflammatory Index (DII) among Young College-aged women. Nutrients 2023; 15. 10.3390/nu15214553 PMC10647813 37960206

[50] Wang J, Wang Y, Guo Z, Lin Z, Jin X, Niu H, Wu Y, Tang L, Hou H (2023). Influence of lifestyle on suboptimal health: insights from a national cross-sectional survey in China. J Glob Health.

