# Supplementary material for: Factors associated with depressive symptoms among cancer patients: a nationwide cross-sectional study
Source: BMC Public Health. 2024 May 29;24:1443. doi: 10.1186/s12889-024-18898-9 (PMC11137935; doi:10.1186/s12889-024-18898-9)
Supplement: Supplementary file 1 — Supplementary Material 1 [file 12889_2024_18898_MOESM1_ESM.docx]

Supplementary Material 1. Multicollinearity diagnosis.

| Domain | Variables | Tolerance | VIF |
| --- | --- | --- | --- |
| Demographic factors | Age | 0.68 | 1.47 |
|  | Sex | 0.771 | 1.296 |
|  | Race | 0.938 | 1.067 |
|  | Marital status | 0.875 | 1.143 |
| Socioeconomic factors | Education | 0.761 | 1.314 |
|  | PIR | 0.741 | 1.349 |
|  | Work type | 0.798 | 1.254 |
|  | Health insurance | 0.894 | 1.118 |
| Behavioural health factors | Smoke | 0.822 | 1.216 |
|  | Sleep | 0.958 | 1.044 |
| Self-rated health status | Health status | 0.983 | 1.017 |
| Comorbidities | Asthma | 0.861 | 1.161 |
|  | Arthritis | 0.879 | 1.138 |
|  | Congestive heart failure | 0.818 | 1.222 |
|  | Coronary heart disease | 0.711 | 1.406 |
|  | Angina | 0.849 | 1.177 |
|  | Heart attack | 0.709 | 1.41 |
|  | Stroke | 0.93 | 1.075 |
|  | Emphysema | 0.836 | 1.196 |
|  | Chronic bronchitis | 0.824 | 1.213 |
| Dietary factors | HEI2015 | 0.48 | 2.081 |
|  | HEI2015_total fruit | 0.309 | 3.233 |
|  | HEI2015_ whole fruit | 0.316 | 3.169 |
|  | HEI2015_total vegetables | 0.723 | 1.383 |
|  | HEI2015_greens and beans | 0.711 | 1.406 |
|  | HEI2015_total protein foods | 0.784 | 1.275 |
|  | HEI2015_seafood and plant proteins | 0.678 | 1.475 |
|  | HEI2015_whole grain | 0.759 | 1.318 |
|  | HEI2015_sodium | 0.698 | 1.434 |
|  | HEI2015_added sugar | 0.731 | 1.369 |
|  | DII | 0.659 | 1.518 |
| Cancer-related factors | Years since cancer diagnosis | 0.945 | 1.058 |
|  | Cancer site | 0.925 | 1.081 |

PIR = poverty impact ratio, HEI2015 = Healthy Eating Index 2015, DII = Dietary Inflammatory Index, VIF = variance inflation factor.
