# Supplementary material for: Factors associated with depressive symptoms among cancer patients: a nationwide cross-sectional study
Source: BMC Public Health. 2024 May 29;24:1443. doi: 10.1186/s12889-024-18898-9 (PMC11137935; doi:10.1186/s12889-024-18898-9)
Supplement: Supplementary file 2 — Supplementary Material 2 [file 12889_2024_18898_MOESM2_ESM.docx]

The regression equation model of depressive symptoms among cancer patients is as follows:

logit(*P*) = 1.465 - 0.733χ_1_ - 1.707χ_2_ - 0.652χ_3_ - 0.957χ_4_ - 0.632χ_5_ + 0.685χ_6_ + 0.561χ_7_ + 0.623χ_8_ + 0.611χ_9_ + 0.673χ_10_ - 1.325χ_11_ + 1.262χ_12_ + 0.584χ_13_ - 0.170χ_14_ - 0.129χ_15_ - 0.669χ_16_ - 0.402* χ_17_ - 1.719χ_18_ - 1.888χ_19_ - 1.946χ_20_ - 1.753χ_21_

**Description**

*P*: The probability of depressive symptoms in cancer patients.

χ_1_ : Cancer patients aged 40-59 years.

χ_2_ : Cancer patients aged ≥60 years.

χ_3_ : Male cancer patients.

χ_4_ : Non-Hispanic black cancer patients.

χ_5_ : Non-hispanic white cancer patients

χ_6_ : Never-married cancer patients.

χ_7_ : Widowed, divorced, or separated cancer patients.

χ_8_ : Cancer patients who are unemployed or actively seeking work.

χ_9_ : Cancer patients who currently smoke.

χ_10_ : Cancer patients who sleep less than 7 hours per day.

χ_11_ : Cancer patients with good self-assessed health.

χ_12_ : Cancer patients with poor self-assessed health.

χ_13_ : Cancer patients with arthritis.

χ_14_ : The total fruit and fruit juice consumption per day of the cancer patients (cup equivalents).

χ_15_ : The consumption per day of dark green vegetables + legumes (beans and peas) in cup equivalents for the cancer patients.

χ_16_ : Patients diagnosed with cancers 5-9 years ago.

χ_17_ : Patients diagnosed with cancers diagnosed within the past 4 years.

χ_18_ : Patients with non-melanoma skin cancer.

χ_19_: Patients with cervix

χ_20_ : Patients with hematological cancers (cancer of the blood, leukemia, lymphoma, and Hodgkin's disease).

χ_21_ : Patients with cancers excluding stomach, lung, colorectal, hepatobiliary and pancreatic, melanoma, non-melanoma skin, skin of unknown type, breast, prostate, bladder, kidney, cervix, uterine, ovary, hematological, and thyroid cancers.
